# Low-Cost Imaging to Quantify Germination Rate and Seedling Vigor across Lettuce Cultivars

**DOI:** 10.3390/s24134225

**Published:** 2024-06-29

**Authors:** Mark Iradukunda, Marc W. van Iersel, Lynne Seymour, Guoyu Lu, Rhuanito Soranz Ferrarezi

**Affiliations:** 1 Department of Horticulture, University of Georgia, Athens, GA 30602, USA; mark.iradukunda@uga.edu (M.I.); mvanier@uga.edu (M.W.v.I.); 2 Department of Statistics, University of Georgia, Athens, GA 30602, USA; seymour@uga.edu; 3 College of Engineering, University of Georgia, Athens, GA 30602, USA; guoyu.lu@uga.edu

**Keywords:** *Lactuca sativa*, chlorophyll fluorescence, crop modeling, embedded computers, image analysis

## Abstract

The survival and growth of young plants hinge on various factors, such as seed quality and environmental conditions. Assessing seedling potential/vigor for a robust crop yield is crucial but often resource-intensive. This study explores cost-effective imaging techniques for rapid evaluation of seedling vigor, offering a practical solution to a common problem in agricultural research. In the first phase, nine lettuce (*Lactuca sativa*) cultivars were sown in trays and monitored using chlorophyll fluorescence imaging thrice weekly for two weeks. The second phase involved integrating embedded computers equipped with cameras for phenotyping. These systems captured and analyzed images four times daily, covering the entire growth cycle from seeding to harvest for four specific cultivars. All resulting data were promptly uploaded to the cloud, allowing for remote access and providing real-time information on plant performance. Results consistently showed the ‘Muir’ cultivar to have a larger canopy size and better germination, though ‘Sparx’ and ‘Crispino’ surpassed it in final dry weight. A non-linear model accurately predicted lettuce plant weight using seedling canopy size in the first study. The second study improved prediction accuracy with a sigmoidal growth curve from multiple harvests (*R*^2^ = 0.88, *RMSE* = 0.27, *p* < 0.001). Utilizing embedded computers in controlled environments offers efficient plant monitoring, provided there is a uniform canopy structure and minimal plant overlap.

## 1. Introduction

Seed banks provide seeds for the next season and are crucial in conserving genes and helping to study plant behaviors. They offer research opportunities to improve food production using different breeding and genetic engineering programs [1,2]. Because seed banks and seed companies require good seed management, it is important to understand how different storage conditions affect seed quality and how that seed quality will translate into healthy plants. Therefore, effective methods are needed to assess seedling vigor and germination rates in seed lots. Quantifying the storage potential (storability) of seeds can help with decisions about appropriate storage conditions and the time of seed release [2,3,4]. In addition, ranking seed lots can ensure that only high-vigor seeds are used, especially when harsh conditions are expected [5].

Since more traditional seed and seedling vigor quantifications are labor-intensive and time-consuming, better technologies based on computer vision are favored [6]. One such method is the seed vigor imaging system (SVIS), where seed quality is quantified from images of the seeds with emerged radicles [7]. Saturated salt accelerated aging (SSAA) with SVIS methods has provided an accurate and rapid seed vigor evaluation in lettuce (*Lactuca sativa*) [8]. However, this method is performed at the seed level and no real-time data can be gathered on the plant performance. A better method combining early seedling growth evaluation and continuous plant growth tracking would provide more information. This method would aid in identifying any deviations from anticipated physiological behavior, which could indicate potential issues or anomalies that could be addressed early. In addition, breeding programs for cultivar development and plant selection take a long time due to the replications required. 

The process can be improved by introducing image-based techniques that are rapid in the phenotyping of plant characteristics, such as identifying plant health traits useful for disease detection (i.e., apple fungal diseases) using spectral imaging and/or chlorophyll fluorescence imaging [9]. When understanding plant performance, great tools like Pulse Amplitude Modulation (PAM) have been crucial in plant sciences. PAM is a non-invasive way to quantify photosynthetic performance of leaves and in some cases a whole plant system [10]. By looking at the light emitted by a photosynthetically active plant, it is possible to deduce how the plant reacts to its surroundings, including biotic and abiotic stress [10]. One simple technique to quickly gauge plant response is chlorophyll fluorescence imaging (CFI), which takes advantage of the plant’s photosynthetic activity [11]. With CFI, special cameras and filters are used to acquire images, and further analysis can provide insights into the leaf or the plant photosynthetic activity and stress responses [11,12,13]. CFI separates the background from photosynthetically active material (plants or algae), giving only pixels of the plant material, which helps to quantify plant size. The plant size and biomass can be predicted using leaf area since more leaf area is associated with more growth due to photosynthesis [14]. Image-based predictions of lettuce growth have been made in other studies [15] using more advanced and accurate models to quantify lettuce growth like artificial neural networks and [16] convolutional neural networks [17]. The downside of most of these techniques is that they are stand-alone tools where plants or the tool must be transported to it to take images, which are then downloaded and analyzed on a computer. Involving multiple steps consumes time, possibly damages plant material, and makes it easy to make other kinds of mistakes. As a solution, cameras could be incorporated into the growing system to collect necessary data without interfering with daily operations. Due to the small space in most vertical farm shelves, there are bright prospects for integration of embedded computers like the Raspberry Pi in various agricultural production systems. With an accuracy of about 98%, embedded computers coupled with deep learning vision techniques could determine the health status of lettuce plants [18]. Because of the lower labor costs associated with their use in controlled environment agriculture, embedded computers promise improved automation, data-driven decision-making, and precision farming [19,20,21]. The embedded computers can be used as an imaging tool due to their adaptability, affordability, and capacity to completely transform agricultural operations by offering accurate and effective plant performance monitoring [21]. In the last decade, these systems have also been utilized in the field despite lighting challenges [22]. Despite great advances in this area, some practical production challenges exist. First, most tools have image acquisition and analysis as separate steps, adding to processing time. Second, by utilizing advanced machine learning models, small-scale researchers and farmers with limited investments and computer skills can find it challenging to utilize them for daily tasks.

Our objective was to utilize imaging techniques to quantify the germination rate and projected canopy size (PCS) of various lettuce cultivars. Then, the acquired PCS was used to predict shoot dry weight. We anticipated that the canopy size, determined through our imaging tools, would be a reliable predictor for seedling dry weight. In the initial investigation, we utilized chlorophyll fluorescence imaging to assess germination rate, uniformity, and seedling vigor across diverse lettuce cultivars exhibiting distinct leaf morphologies. In the next phase, we applied the insights gained to integrate embedded computers into a vertical farm and a walk-in growth chamber with shelves to acquire real-time data that can be accessed remotely. Throughout both studies, we hypothesized that differences in canopy behaviors would be evident among various lettuce cultivars and that canopy size can predict shoot dry weight.

## 2. Materials and Methods

### 2.1. Location and Environmental Conditions

The experiment was conducted at the University of Georgia (College of Agricultural and Environmental Sciences, Department of Horticulture, Horticultural Physiology and Controlled Environment Agriculture laboratories), in Athens, GA, USA (latitude 33°57′26.676″ N, longitude 83°22′36.48″ W). The plants were grown in a walk-in growth chamber with three stacked shelves. The conditions for the entire study were maintained at 25 °C temperature, 70% relative humidity, 0.91 kPa vapor pressure deficit, 800 mg/L of carbon dioxide, light intensity of 250 µmol·m^2^·s^−1^, and 16 h of photoperiod (12 a.m. to 4 p.m.), resulting in a daily light integral of 14.4 mol·m^−2^·d^−1^. Daily irrigation was provided using a 15N-2.2P-12.5K fertilized water solution with 100 mg/L of nitrogen (15-5-15 Ca-Mg Professional LX; J.R. Peters Allentown, PA, USA) through an automated ebb-and-flow subirrigation system activated for 5 min daily.

### 2.2. Experimental Setup for Chlorophyll Fluorescence Imaging Study

For the first study, we selected nine green-colored lettuce cultivars: ‘Bauer’, ‘Crispino’, ‘Cristabel’, ‘Grazion’, ‘Ilema’, ‘Little Gem’, ‘Muir’, ‘Rex’, and ‘Sparx’ (Jonny’s Selected Seeds, Winslow, ME, USA) with distinct growth patterns. For each cultivar, we sowed 12 pelleted seeds (one seed per cell) in 3 × 4 tray cells (DPS98 Round Propagation Sheet; The HC Companies, Twinsburg, OH, USA). For the growing medium, we used a soilless mix (MM830-F3B Professional Mix; Sungro Horticulture, Agawam, MA, USA). To prevent algae growth, we covered the surface of the substrate with turface (Profile^®^ Products, Buffalo Grove, IL, USA) which keeps the surface dry. We created five replications by randomly arranging nine sub-trays of 3 × 4 cells in two 50.80 × 21.59 × 6.35 cm trays with drainage holes (606 R8 8520 Slim Jim Vacuum Flat; The HC Companies, Twinsburg, OH, USA). The trays were placed on the shelves inside the chamber. Clear plastic domes were placed over the trays, which were removed once germination became visible (approximately 3 days after seeding).

At 14 days post-sowing, we transplanted half (six) of the seedlings randomly selected from each cultivar into 15.24 cm deep black pots (SVD-550-square plant pots; T.O. Plastics, Clearwater, MN, USA), creating four replications. The remaining seedlings were harvested and dried, and their dry weight was recorded. At 40 days post-sowing, mature lettuce was harvested, and dry weight was recorded. All dry weight was recorded using a scale (Analytical Balance PR224; Ohaus Corporation, Parsippany, NJ, USA). The experiment was a completely randomized design with 12 plants in 3 × 4 tray cells representing an experimental unit. There were nine cultivar variations in predicted canopy size and dry weight over five replications during the seedling stage and four replications of six plants after transplantation.

### 2.3. Image Acquisition Using Chlorophyll Fluorescence Imaging

For image acquisition in the first study, trays had to be moved from the chamber to the imaging system and back. To take images, we placed 3 × 4 cell blocks (containing 12 seedlings) at the center of the imaging system (chlorophyll fluorescence imaging system within a sealed tent and a reflective interior [12,23]). Blue light-emitting diodes (LEDs) (Pro 650e; LumiGrow, Emeryville, CA, USA) were activated to trigger photosynthesis, and a camera (Chameleon^®^ 3; FLIR, Wilsonville, OR, USA) with a long-pass filter (SP700-R45X2; Midwest Optical Systems, Inc., Palatine, IL, USA) was employed to capture light emitted from plant material in the red/infrared spectrum (650–740 nm). The camera was connected to a computer via a USB cable, and images were captured using commercial software (Spinnaker SpinView 2.3.0.77; Teledyne FLIR, Wilsonville, OR, USA). The image acquisition settings included the following: Acquisition Mode: Continuous, Acquisition Frame Rate: 1.01, Exposure Compensation Auto: Continuous, Exposure Mode: Timed, Exposure Auto: Off, Exposure Time: 99860.19, Gain Auto: Off, Gain: 10, Black level: 0, and Device Link Throughput Limit: 9504000.

The acquired images were saved, downloaded, and subsequently analyzed using Python code. Images were captured on Mondays, Wednesdays, and Fridays. After each imaging session, trays were randomly repositioned within the walk-in growth chamber.

### 2.4. Embedded Computer Setup

We conducted a second study under the same conditions to incorporate imaging technology in the growing system. In this study, we configured eight embedded computer devices (Raspberry Pi 4 Model B 8GB RAM; Raspberry Pi Foundation, Cambridge, UK) with 256/128GB SD cards for storage (Extreme microSDXC UHS-I Memory Card; SanDisk Corporation, Milpitas, CA, USA). Each device was equipped with a camera (Raspberry Pi Camera Module 3 Wide; Adafruit Industries, Varick, NY, USA), connected to power and ethernet cables, and enclosed in a case. These devices were mounted on the top shelves overlooking the trays. To ensure minimal disturbance of other daily activities in the area, all embedded computers were remotely accessible using the PC’s remote desktop connection app, and all data were synchronized to the cloud (OneDrive; Microsoft Corporation, Redmond, WA, USA).

### 2.5. Experimental Setup for Embedded Computers Study

**Figure 1:**
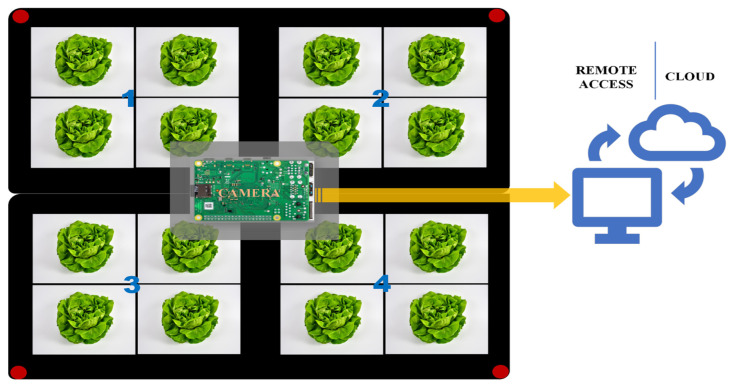
Illustration of the experimental setup representing one replication. (1–4): Positional identifiers of groups of plants belonging to different cultivars (arrangements are randomized in each application). (Camera): An embedded computer with a camera installed. Collected data are uploaded to the cloud at one-minute intervals.

We selected four green-colored lettuce cultivars: ‘Bauer’, ‘Crispino’, ‘Cristabel’, and ‘Muir’ (Jonny’s Selected Seeds, Winslow, ME, USA). We sowed 32 seeds of each cultivar in black/green pots, distributed across eight replications (each embedded computer device represents one replication). The reason for many replications was to offset issues with potential device malfunction. Each replication consisted of sixteen pots, with four pots allocated to each cultivar, following a random design structure. Two standard trays were positioned side by side to create a square, with four pots on the edge of each tray. Red/blue pins were placed at the four corners of this square for subsequent segmentation, allowing multiple treatments within one image (Figure 1). We harvested the plants three times from each cultivar to measure dry weight over time: the first at 14 days post-sowing (two plants), the second at 21 days post-sowing (one plant), and the third at 33 days post-sowing (one plant). We carefully selected plants for each replication to minimize overlap and ensure the camera adequately captured each plant. There were four cultivar variations in predicted canopy size and dry weight over eight replications.

### 2.6. Image Acquisition Using Embedded Computers

**Figure 2:**
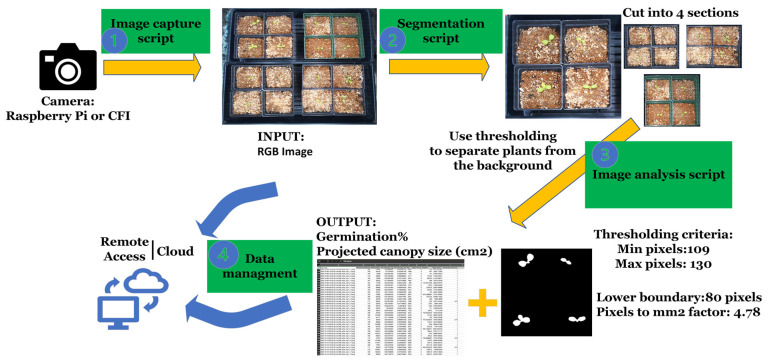
Flowchart illustrating the steps from image acquisition to cloud data uploading. Step 1 involves manual image capture through chlorophyll fluorescence imaging or automated acquisition using a Python script on embedded computers. In Step 2, a segmentation script divides the captured colored images (RGB) into four sections, each representing different treatments. Step 3 encompasses the analysis of segmented images by the script, which saves the results in a .CSV file. Finally, in Step 4, all data are uploaded to the cloud, facilitating remote access and management.

To enhance automation, images were scheduled to be captured four times each day (00:00, 05:00, 12:00, and 15:00 h), and the results were averaged daily. Two minutes after image acquisition, the next script ran to create perfect squares using the red/blue dots placed on the trays’ corners. This allowed for division into four equal parts, producing four individual images representing cultivars within one replication. This feature enabled multiple treatments within one image (same tray) but added to the processing time. The image analysis script ran four minutes after segmentation, generating analyzed images and meaningful data stored in .CSV files. Concurrently, another script uploaded images, .CSV files, and plots to the cloud every minute, ensuring data retrieval even if one of the steps encountered an issue. At the experiment’s end, .CSV files were consolidated from all eight devices to facilitate statistical analysis. The flowchart of the steps involved can be found in Figure 2. Relevant scripts are available for viewing and download at [24].

### 2.7. Image Analysis

**Figure 3:**
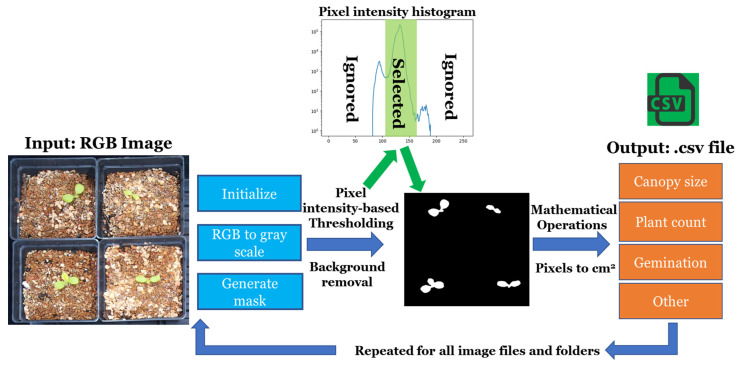
Flowchart illustrating the analysis program logic. Initially, the program reads a colored image (RGB) file and converts it into grayscale to facilitate image manipulations. Subsequently, a pixel-intensity histogram of the entire image is generated. The program then selects pixels corresponding to the plant material while disregarding those representing the background, creating a binary image where white represents the plant and black indicates the background. Using mathematical equations, the binary image is utilized to extract pixel information, including area and the number of observed plants. Furthermore, additional equations are applied to convert pixels into cm^2^, calculate germination percentage, and derive other relevant information. Finally, all the data are stored in a .CSV file, which is subsequently used for further analysis.

We analyzed images using Python-based code developed by the author in collaboration with the University of Georgia’s Horticultural Physiology and Controlled Environment Agriculture Labs [24]. This code generated pixel-based measurements converted to cm^2^ referencing a known object’s distance captured before each image acquisition session using Equation (1):

(1) $$y=\frac{x}{{\left(\frac{a}{b}\right)}^{2}}$$

where *y* is the PCS (cm^2^), *x* is the area of plant material in the image (pixels), *a* is the length of a known object (pixels), and *b* is the length of a known object (cm). The logic of the analysis program can be found in the illustration shown in Figure 3.

Analyzed images were saved, and the resulting data were stored in .CSV files for further analysis using statistical software (R Studio version 4.3.1; R Foundation for Statistical Computing, Vienna, Austria) and Python (Python 3.11; Python Software Foundation, Wilmington, DE, USA), facilitating visualization and statistical assessments. These conversions and basic statistical calculations were performed automatically within the Python script in the case of the second study using the embedded computer.

### 2.8. Measurements

Using image analysis, we counted the number of plants germinated to calculate germination percent. The area in pixels was transformed into area in cm^2^ using calibration as described above. That area and number of plants were used to calculate the average PCS per plant. After each harvest, plants were dried to collect the average shoot dry weight per plant.

### 2.9. Statistical Analysis and Modeling

One-way analysis of variance (ANOVA) and Tukey’s HSD were conducted to determine the significant differences in PCS and dry weight amongst cultivars. A coefficient of determination (*R*^2^), root mean square error (*RMSE)*, and *p*-value (*p*) were used to evaluate the performance of the regression models in predicting dry weight from PCS. In the data exploration stage, the dry weight data violated normality and homoscedasticity (constant residual error variance) assumptions. That is not surprising since we combined data from all the days (very small seedlings and bigger plants at the final harvest), which made linear regressions inappropriate. We log-transformed the response variable (shoot dry weight) and used a non-linear model to fit the curve. To accurately model the growth behavior of the plants, we used sigmoidal curves given by Equation (2):

(2) $$y=\frac{a}{1+e^{-b*\left(x-c\right)}}$$

where *y* is the shoot dry weight (the dependent variable), *x* is the PCS (the independent variable), *a* is the maximum shoot dry weight achievable, *b* is the steepness of the curve controlling the growth rate, and *c* is the midpoint of the curve indicating the PCS at which the growth rate is half of the maximum.

## 3. Results

### 3.1. Projected Canopy Size over Time

**Figure 4:**
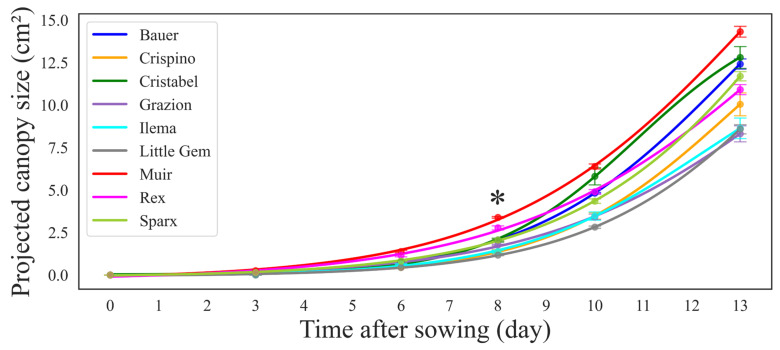
The curves showing the relationship between the projected canopy size (PCS) of lettuce (*Lactuca sativa*) seedlings of each cultivar obtained by chlorophyll fluorescence imaging and time (days after sowing). PCS data were collected from sowing on days 3, 8, 10, and 14 (at transplantation time). Each data point and the error bars indicate the mean and standard deviation of 24 seedlings of each day in each cultivar. * indicates a significant difference between cultivars using Tukey’s HSD at α = 0.05.

The PCS increased over time for all lettuce cultivars (Figure 4). In the initial phase, no germination occurred until day two, followed by a gradual increase in PCS from day three to five, with exponential growth thereafter (Figure 4). Notably, cultivars with higher initial PCS values continued to exhibit larger sizes throughout the 14-day germination period with some variations. The cultivar ‘Muir’ showed big size and fast growth throughout the germination period, while the cultivar ‘Little Gem’ was the smallest with a slow growth curve. However, significant differences in PCS were observed only on day eight after sowing (Tukey’s HSD, *p* = 0.03).

**Figure 5:**
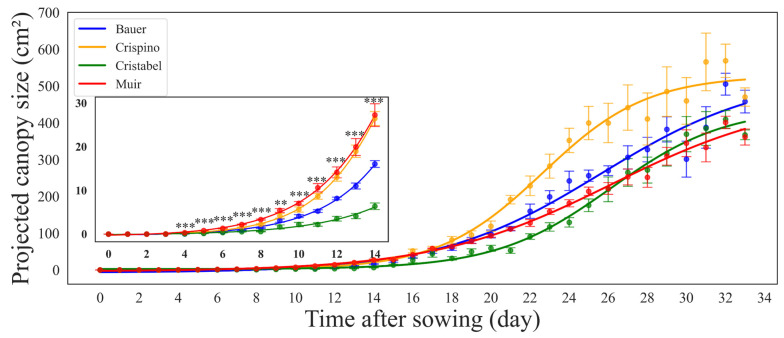
The curves showing the relationship between the projected canopy size (PCS) of lettuce (*Lactuca sativa*) seedlings of each cultivar obtained by embedded computers. PCS data were collected every day from sowing to 14 days. Each data point and the error bars indicate the mean and standard deviation of 32 seedlings of each day in each cultivar. Insert displays PCS for seedling stage (initial 14 days after sowing). **, *** indicate significant differences between cultivars during the initial 14 days using Tukey’s HSD at α ≤ 0.01, and 0.001, respectively.

In the second study with embedded computers, a similar trend in PCS increase was observed (Figure 5). On average, the cultivar ‘Muir’ showed a big size and fast growth throughout the germination period, while the cultivar ‘Cristabel’ was the smallest with a slow growth curve. Starting from day four after sowing, there were significant differences in PCS (Tukey’s HSD, *p* < 0.001) on all analyzed days and day nine (Tukey’s HSD, *p* = 0.002). At 16 days after sowing, cultivar performance changed, where ‘Crispino’ surpassed all of them, and the cultivar ‘Muir’ finished with a lower PCS.

### 3.2. Germination Curves

**Figure 6:**
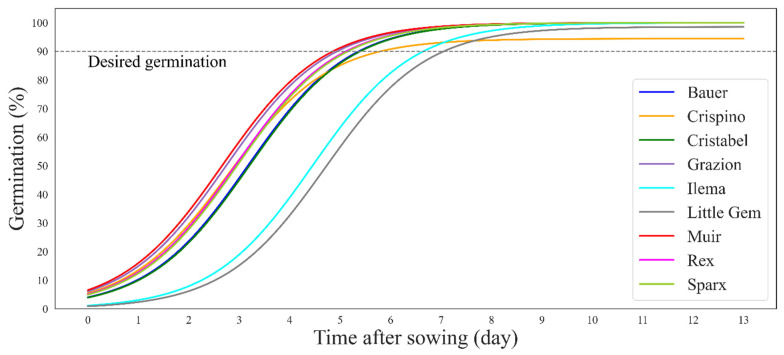
Sigmoidal curves showing germination percentage over time colored by cultivar. Germination percentage was calculated from data obtained by chlorophyll fluorescence imaging. The grey dashed line shows the desired germination (90%), and where the curve does not reach the line means poor germination.

**Figure 7:**
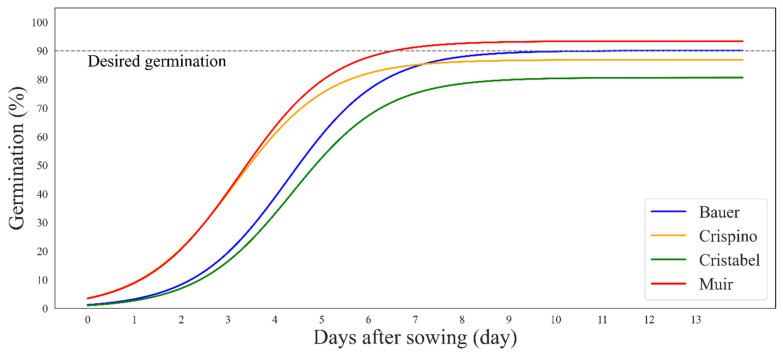
Sigmoidal curves showing germination percent over time colored by cultivar. Germination percentage was calculated from data obtained by embedded computers. The grey dashed line shows the desired germination (90%), and where the curve does not reach the line means poor germination.

Germination percentage over time was recorded, revealing that all cultivars reached 90% germination within days (Figure 6 and Figure 7). One of the helpful features of this germination evaluation tool is that it indicates when a desired germination has been reached. We assumed 90% as the desired germination (this value can be adjusted). All cultivars reached 90% germination within seven days (Figure 6). The cultivar ‘Little Gem’ had the slowest germination, while the cultivar ‘Muir’ had the fastest. It only took four days for cultivars ‘Muir’ and ‘Grazion’; five days for cultivars ‘Bauer’, ‘Crispino’, ‘Cristabel’, ‘Rex’, and ‘Sparx’; six days for ‘Ilema’; and seven days for ‘Little Gem’ to germinate.

For the second study with embedded computers, the cultivar ‘Muir’ had the fastest germination while the cultivar ‘Cristabel’ had the slowest and worst germination (Figure 7). It took six days for the cultivar ‘Muir’ to reach 90% germination and eleven days for the cultivar ‘Bauer’, while cultivars ‘Crispino’ and ‘Cristabel’ did not reach 90% germination as they did in the first study. The differences in cultivar germination between the two studies were likely due to differences in seed lots and the number of plants used in both studies.

### 3.3. Shoot Dry Weight

**Figure 8:**
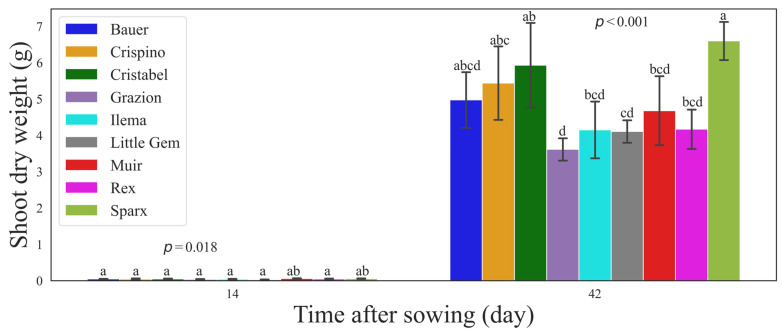
Shoot dry weight of lettuce (*Lactuca sativa*) cultivars collected 14 days after sowing (transplantation time) and 42 days after sowing (final harvest). Each bar and the error bars indicate the mean and standard deviation of 24 seedlings in each cultivar. Values followed by the same letter in each harvest are not significantly different, according to Tukey’s HSD at α = 0.05.

Significant cultivar variations were observed (*p* = 0.018) in dry weight collected on day 14 (before transplantation) and final shoot dry weight on day 42 (*p* < 0.001) (Figure 8). Fourteen days after sowing, the cultivar ‘Muir’ had the highest shoot dry weight of 0.059 g. On the other hand, the cultivar ‘Sparx’ had the highest shoot dry weight of 6.598 g at the final harvest (day 42). The average weight of cultivars ‘Bauer’, ‘Crispino’, ‘Cristabel’, ‘Grazion’, ‘Ilema’, ‘Little Gem’, ‘Muir’, ‘Rex’, and ‘Sparx’ was 0.048, 0.045, 0.049, 0.037, 0.041, 0.029, 0.059, 0.051, and 0.056 g on day 14 and 3.614, 4.150, 4.107, 4.678, 4.170, and 6.598 g on day 42, respectively. Multiple comparisons revealed significant differences between cultivars (Tukey’s HSD, α = 0.05), where values that share similar letters are not significantly different (Figure 8).

**Figure 9:**
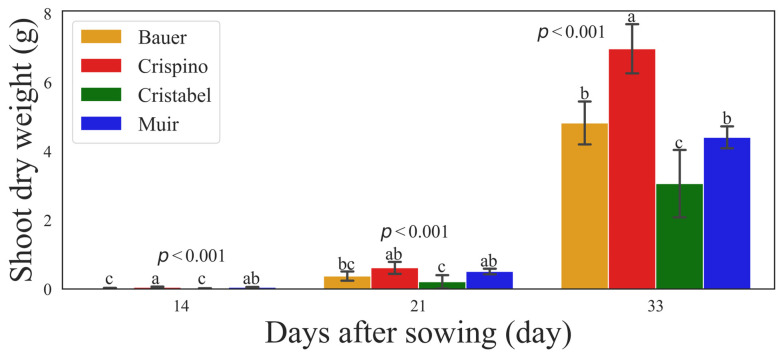
Dry weight collected 14, 21, and 33 days after sowing. Each bar and the error bars indicate the mean and standard deviation of 32 seedlings in each cultivar. Values followed by the same letter in each harvest are not significantly different, according to Tukey’s HSD at α = 0.05.

In the second study using embedded computers, significant cultivar variations in shoot dry weight were observed on days 14, 21, and 28 (*p* < 0.001) (Figure 9). The cultivar ‘Crispino’ had the highest shoot dry weight of 0.059 g 14 days after, 0.615 g on 21 days, and 6.598 g at 28 days after sowing. The average weight of cultivars ‘Bauer’, ‘Crispino’, ‘Cristabel’, and ‘Muir’ was 0.025, 0.052, 0.018, and 0.05 g on day 14; 0.375, 0.615, 0.211, and 0.512 g on day 21; and 4.824, 6.979, 3.061, 4.405 g on day 28, respectively. Multiple comparisons revealed significant differences between cultivars (Tukey’s HSD, α = 0.05), where values that share similar letters are not significantly different (Figure 9).

### 3.4. PCS and Shoot Dry Weight

Using one-way ANOVA, we found that the PCS of 14-day-old seedlings is a strong explanatory variable of the final dry weight (*F* (1,18) = 44.264, *p* < 0.001). Cultivar was found to be significant (*F* (8,18) = 9.476, *p* < 0.001), though there was no evidence of significant interaction between the PCS of the seedlings and the cultivar (*F* (8,18) = 0.98, *p* < 0.482) (Table 1).

**Figure 10:**
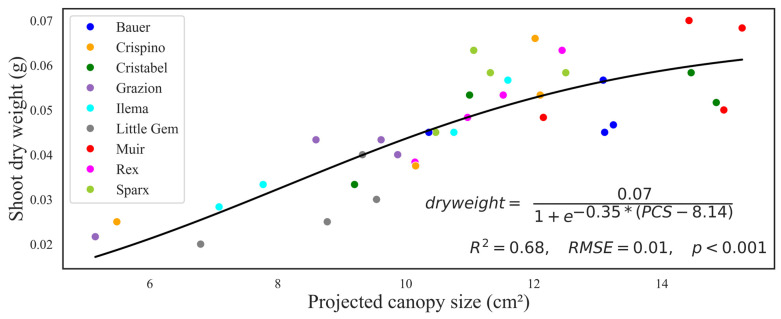
The relationship between seedling projected canopy size (PCS) and corresponding seedling shoot dry weight colored by cultivar. The black solid line shows a sigmoidal regression with the regression equation, regression summaries of coefficient of determination (*R*^2^), root mean square error (*RMSE*), and *p*-value (*p*).

There was a positive correlation between the average seedling canopy size and the average seedling dry weight. A general non-linear sigmoidal model encompassing all cultivars in predicting seedling dry weight from PCS performed adequately (*R*^2^ = 0.68, *RMSE* = 0.01, *p* < 0.001) (Figure 10). The model performance issue was fixed in the second study by using more data. From the regression equation, the maximum dry weight was 0.07 g, with a growth rate of 0.35 g/cm^2^. A PCS of 8.14 cm^2^ was required to reach half of the maximum growth rate.

**Figure 11:**
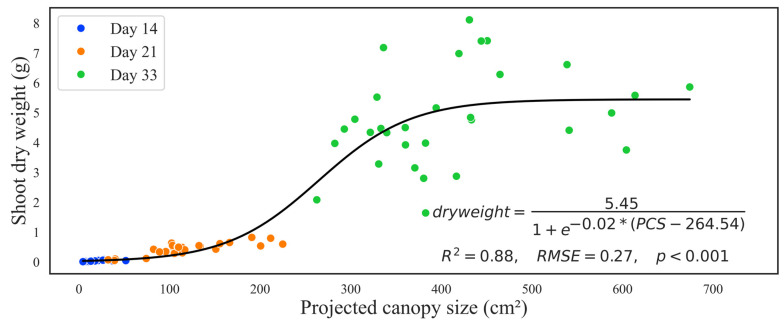
The relationship between projected canopy size (PCS) and corresponding shoot dry weight colored by days of harvest (to collect shoot dry weight). The black solid line shows a sigmoidal regression with an equation, regression summaries of coefficient of determination (*R*^2^), root mean square error (*RMSE*), and *p*-value (*p*). Canopy size data obtained by embedded computer devices.

For the second study with embedded computers, combining canopy size data with all three harvests, canopy size proved to be a better predictor of dry weight (t = 18.656, *p* < 0.001), while cultivar had no significant effect at (t = 0.199, *p* = 0.843). A sigmoidal regression model (generalized for all three harvests and cultivars) showed a strong positive relationship between PCS and the shoot dry weight (*R*^2^ = 0.88, *RMSE* = 0.27, *p* < 0.001) (Figure 11). There was high variability due to cultivar on day 33, which means that the strong *R*^2^ was heavily influenced by days 14 and 21. From the regression equation, the maximum dry weight was 5.45 g, with a growth rate of 0.02 g/cm^2^. A PCS of 264.54 cm^2^ was required to reach half of the maximum growth rate.

## 4. Discussion

As plants grow, they increase in size due to cell division and expansion, which is why we saw an exponential increase in PCS in all cultivars during the first 14 days (Figure 4). As expected, the increase in PCS over time (growth rate) was different amongst the cultivars studied. For example, the cultivar ‘Muir’ showed consistently larger PCS than other cultivars at the seedling stage (Figure 4). This is not surprising, as ‘Muir’ grows slowly and outward to allow a better imaging view even in early stages where seedlings are prone to flopping. ‘Muir’ is also well known for its hardiness and can do well in diverse conditions [25]. This is why ‘Muir’ also showed a better germination rate (Figure 6). On the other hand, ‘Little Gem’ performed poorly in PCS (Figure 4), and had a much slower germination rate compared to the cultivar ‘Muir’ (Figure 6). A similar study found that ‘Little Gem’ does not do well in hydroponics under different light sources among 26 cultivars studied [26]. Here, we just compared the best- and the worst-performing cultivars. In the following paragraphs, we provide a complete picture of what happened at different time points and the implications.

The question is, does the best plant at the seedling stage become the best at the final stage regarding dry weight? Let’s look at cultivars with known good performance. A study found that the cultivar ‘Sparx’ has superior marketable yield compared to other romaine lettuces under heat stress [17], which explains why it had the best final dry weight. The same observations were made for the ‘Muir’ cultivar [25]. In the first study, the cultivar ‘Muir’ had the highest dry weight on day 14, but ‘Sparx’ surpassed it by day 42 (Figure 8). At the early stage, these plants were very close in their dry weight, where ‘Muir’ had 0.003 g more than ‘Sparx’. However, at the harvest stage on day 33, ‘Sparx’ was better by 1.92 g, which implies that the growth performance differences among cultivars at the seedling stage are not necessarily maintained through the growth cycle. These cultivars differ in leaf morphology, head formation, and growth rate. ‘Muir’ is a slow-growing cultivar with wavy leaves, while ‘Sparx’ grows fast with longer leaves. ‘Muir’ seemed to have high PCS in the early stages simply because it grows more outwards and allows better imaging, while ‘Sparx’ grows upward with more erect leaves, which makes the canopy difficult to project with a top-view imaging system. One issue with this study is that we focused on PCS at the seedling stage for the initial data analysis. After observing inconsistencies with shoot dry weight, we included full PCS data growth throughout the growth cycle in the second study. In doing that, it was possible to compare complete PCS data and dry weight side by side.

In the second study, we compared ‘Muir’ with another good-performing cultivar, ‘Crispino’. During the first 14 days after sowing, ‘Muir’ did better than other cultivars in PCS (Figure 5) and germination (Figure 7). On the other hand, the cultivar ‘Crispino’ outperformed others by having the highest dry weight at all stages (Figure 9), even with poor initial germination (Figure 7). Similar results were found when lettuce cultivars were compared in raised beds with different kinds of mulches, and ‘Crispino’ had the highest yield [27]. Looking at the complete PCS, it is clear that starting from day 16, the cultivar ‘Crispino’ started showing a much faster growth rate than ‘Muir’ until the end. That is because ‘Crispino’ has long, loose leaves that begin to flop and occupy more space as they grow heavy, while cultivars like ‘Muir’ remain more uniform and compact. The contradictions between the top view size and the dry weight are worsened when the plant leaves overlap or the leaves’ surface is inconsistent, like in ‘Crispino’. So, it is not appropriate to draw comparative conclusions about cultivar performance based on PCS alone. The PCS relationship to dry weight depends heavily on the leaf structure (leaf area and thickness) and the head compactness of the lettuce. For example, leaf area alone has proved to be an unreliable predictor of plant biomass in *Arabidopsis* as the plant matures and energy is spent in other parts of the plant [28]. Therefore, more accurate comparisons are achieved when cultivars grow uniformly outward with similar canopy structures and maturity times. Due to space in our growth chamber and the limited camera view, we harvested all plants simultaneously, ignoring possible differences in maturity timing. In addition, the cultivar ‘Muir’ is slow-growing, and growing it longer could have yielded different final dry weight results.

The sigmoidal regression model revealed that the relationship between PCS and shoot dry weight is not linear (Figure 10). Due to the lack of PCS for the final day in the first study, we could not fit the full curve, and the results were not conclusive, but this problem was fixed in the second study. In both studies, we confirmed our hypothesis that PCS is a stronger predictor for dry weight than cultivars (Table 1 and Figure 11). The cultivar variations increased with time, and day 33 had the most variations. Similar results have been found in another study when, after day 29, the graph became unstable as more plant area grew out of the camera view [15]. The easiest solution would be to model days separately. Nonetheless, our technique can be used to select more vigorous plants at early stages before plants are too big or leaves thicker. Other studies have been able to predict plant biomass from canopy size in lettuce [12], cereals [29], and *Arabidopsis* [28]. The leaf area is a great factor in determining growth as more leaves capture more light that can be used in photosynthesis and related processes. Embedded computers equipped with special cameras accurately quantified the leaf area of lettuce in field studies [22]. The sigmoidal regression model demonstrated that an S-shaped curve is a better model for plant growth. Using simple linear regression and evaluating the models using *R*^2^ is always tempting in regression analysis. However, when working with organisms like plants, the growth cannot be linear, and the data violate basic assumptions of normality and homoscedasticity. The authors recommend exploring other non-linear models appropriate to plant behavior and growth rates in different situations [30,31]. Enhancing resources and leveraging advanced modeling expertise could lead to developing a sophisticated model incorporating additional plant attributes such as height, age, and environmental variables. This comprehensive approach has the potential to significantly enhance the accuracy of predictions [21]. Leaf area was used to determine the growth stage of lettuce plants using artificial neural networks [16]. Similarly, a convolutional neural network method accurately predicted lettuce growth features like leaf area and shoot biomass in the greenhouse [17]. Limitations of our technique include physical space, the position of the camera, and image analysis stages. The distance between the camera and the plants is reduced when plants grow more vertically, forcing us to harvest plants earlier than the normal harvest time. Though more horizontal growth is desirable for more canopy exposure, too much could lead to plants touching. When plants touch, it becomes harder to separate individual plants, leading to underprediction of dry weight as some plant material is hidden from the camera view. Taking images in two dimensions (top view) hides important information, including height, which reduces the prediction accuracy. That said, there is great progress in utilizing smart tools to monitor plant performance. A portable imaging Raspberry Pi camera could accurately detect the disease status of lettuce [18]. However, as with our technique, the authors admit that they trained on iceberg lettuce, and nothing could be said about other plants [18]. Future studies could combine artificial intelligence modules with the practical space adaptability of our technique to make a more robust tool that requires less user intervention and features the availability of real-time data.

## 5. Conclusions

These studies showed a positive correlation between canopy size and dry weight, underscoring the importance of early canopy development in predicting plant yield. The results highlight the influence of cultivar selection on germination, growth, and final biomass. In comparing cultivars, there is the potential of finding contradicting canopy size and dry weight results at different time points. For instance, ‘Muir’ showed overall high PCS and dry weight at the seedling stage, but other cultivars like ‘Sparx’ and ‘Crispino’ surpassed it by the time they reached maturity. These variations were simply due to the cultivar differences in canopy structure. Though our goal was not to select the best cultivar, our study shows that canopy size and early germination behaviors can be used to gain quick insights into plant performance and to find any notable differences in growth behavior. However, with too much variation in the canopy behavior of those cultivars, other parameters should be considered. Ensuring the accuracy of plant dry weight prediction based on canopy size hinges on the plant’s ability to exhibit consistent and evenly distributed growth patterns in two dimensions. Since we observed many variations at maturity, our technique would work better with young and medium-sized plants. This approach minimizes leaf overlap instances and maximizes leaf surface exposure. Transitioning from CFI to an incorporated embedded computer system saved more time and provided real-time data on plant performance that could be accessed remotely. Understanding how plants are doing in real-time is valuable since it is possible to notice and fix potential problems early on. The challenges of reduced accuracy as plants grow bigger can be fixed by being proactive and employing more image analysis and prediction models that are being developed as technology develops.

## Figures and Tables

**Table 1:** Analysis of variance (ANOVA) table to determine predictors significantly affecting final shoot dry weight of lettuce (*Lactuca sativa*) cultivars. *** indicate significant effects at *p* ≤ 0.001. *df* = degrees of freedom, *RMSE* = root mean square error, *F* = *F* statistic, and *p* = *p*-value.

Parameter	Descriptive Statistics			
	*df*	*RMSE*	*F*	*p*
Seedling Canopy Size	1	3.75	44.264	<0.001 ***
Cultivar	8	1.73	9.476	<0.001 ***
Seedling Canopy Size × Cultivar	18	0.56	0.980	0.482

## Data Availability

Dataset available on request from the authors.
